# SPP1 promotes radiation resistance through JAK2/STAT3 pathway in esophageal carcinoma

**DOI:** 10.1002/cam4.4840

**Published:** 2022-05-20

**Authors:** Meijie Wang, Xiaozheng Sun, Huixian Xin, Zhihua Wen, Yufeng Cheng

**Affiliations:** ^1^ Department of Radiation Oncology, Qilu Hospital, Cheeloo College of MedicineQilu Hospital, Cheeloo College of Medicine Shandong University Jinan China; ^2^ Laboratory of Basic Medical Sciences, Qilu Hospital, Cheeloo College of Medicine Shandong University Jinan China

**Keywords:** esophageal carcinoma, JAK2, radiation resistance, SPP1, STAT3

## Abstract

**Background:**

Therapeutic resistance to radiotherapy is one of the major obstacles in clinical practice that significantly affect the therapeutic efficiency and prognosis of human esophageal carcinoma (ESCA). Thus, it is critical to understand the molecular mechanisms of radiation resistance in ESCA. Secreted phosphoprotein 1 (SPP1) plays an essential role in various human cancers, but its role in radiation resistance remains unclear.

**Method:**

Cell culture and transfection; Cell Counting Kit‐8 (CCK‐8) assays; EdU incorporation assays; Patient sample collection and medical records review; Transwell assays; Colony formation assays; Wound healing assays; Western blot; Immunofluorescence; Immunohistochemistry; Irradiation; Flow cytometry; Animal studies; Human Apoptosis Array Kit; Bioinformatics.

**Result:**

In the current study, we reported the novel phenomenon that radiation‐treated human ESCA cells upregulated SPP1 expression, which in turn contributed to the ESCA resistance to radiotherapy. We also reported the tumor‐promoting effect of SPP1 in ESCA systematically and comprehensively. Furthermore, subsequent studies by knocking down or overexpressing SPP1 in human ESCA cells showed that SPP1 could facilitate the repair of DNA damage and the survival of tumor cells post‐radiation in ESCA, which might contribute to the development of radiation resistance during the radiotherapy process. More detailed investigations on the downstream molecular pathway suggested that radiation could increase the phosphorylation level of JAK2 and STAT3 by increasing SPP1 expression. Further in vivo validation using a mouse ESCA xenograft model showed that SPP1 overexpression significantly increased tumor volume while either SPP1 knockdown or pharmacological inhibition of the JAK2‐STAT3 pathway reduced tumor volume in a synergistic manner with radiotherapy.

**Conclusion:**

Collectively, these findings suggested that the SPP1/JAK2/STAT3 axis is a critical player in ESCA progression and radiation resistance, which is a potential therapeutic target for combined therapy with the standard radiotherapy regimen to improve curative effect and increase patients' survival with ESCA.

## Graphical abstract

We are the first to found that radiation could increase phosphorylation level of JAK2 and STAT3 by increasing SPP1 expression, and identified that the SPP1/JAK2/STAT3 axis is a critical player in ESCA progression and radiation resistance, which is potential target for combined therapy to improve curative effect and increase patients’ survival with ESCA.

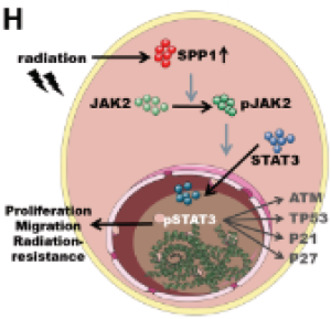

## 1 INTRODUCTION

Esophageal carcinoma (ESCA) is the seventh most common cancer and the sixth leading cause of cancer‐related deaths worldwide.^1^ Despite the recent development in comprehensive treatment regimens, the prognosis of ESCA remains poor.^2^, ^3^, ^4^ Radiotherapy is one of the standard treatments for ESCA, which can efficiently kill cancer cells, alleviate symptoms and prolong patients' survival.^5^, ^6^, ^7^, ^8^ However, ESCA tissue frequently develops radiation resistance during radical, perioperative, or palliative radiotherapy treatments, which ultimately results in treatment failure and has become one of the major obstacles to ESCA treatment in clinical practices.^9^, ^10^, ^11^ Hence, it is urgent to understand the molecular mechanisms underlying the development of radiation resistance in ESCA and subsequently identify the molecular targets for radiation sensitization to develop novel therapeutic approaches.

Secreted phosphoprotein 1 (SPP1), also known as osteopontin, belongs to a family of secreted acidic proteins.^12^ Full‐length SPP1 can be modified by thrombin cleavage and exposes an epitope for integrin receptors of α4β1, α9β1, and α9β4, upon binding these receptors, cells use several signal transduction pathways to elicit immune responses.^13^, ^14^ SPP1 is commonly expressed in cardiac fibroblasts, osteocytes, macrophages, smooth muscle, and endothelial cells,^15^, ^16^, ^17^, ^18^ and regulates many biological processes such as biomineralization, bone remodeling, chemotaxis, cell activation, and apoptosis.^12^, ^19^, ^20^, ^21^, ^22^, ^23^ Under pathological conditions, SPP1 was found to be highly expressed in a variety of types of cancers^24^, ^25^ and was considered oncogenic by contributing to tumor proliferation, invasion, and stem‐like behavior, which all facilitate tumorigenesis and progression.^20^, ^25^, ^26^, ^27^ Recently, SPP1 has been implicated as a prognostic and diagnostic marker for certain cancer types, such as gastric cancer, liver cancer, and breast cancer.^28^, ^29^, ^30^, ^31^, ^32^ As for the current research on SPP1 in ESCA, Song et al., Feng et al. and Tang et al. reported SPP1 as a hub gene by integrated bioinformatics analysis, which exhibited markedly increased expression in ESCA samples compared with non‐cancerous samples.^33^, ^34^, ^35^ In addition, Wang et al., Zhang et al., and Li et al. analyzed the prognostic value of SPP1 and reported that SPP1 is associated with worse disease‐free survival in ESCA through TCGA or other databases with no experiment verification,^36^, ^37^, ^38^ while the diagnostic value of SPP1 still needs to be verified by tissue specimens, which is part of the research we need to do. What is more, there are few studies on the function of SPP1 in ESCA, Jules Lin et al. found SPP1‐b‐type significantly enhanced ESCA cells' migration ability by adding exogenous SPP1.^39^ Nevertheless, the specific function and role of SPP1 in ESCA, especially the role involved in radiation resistance, remains largely unstudied, which motivated our current study to investigate the function of SPP1 in ESCA.

The JAK–STAT signaling pathway consists of several major proteins including JAK1, JAK2, JAK3, STAT1, STAT2, STAT3, and STAT5.^40^ It is heavily involved in basic cell biologies such as cell proliferation, differentiation, and survival.^41^ The JAK–STAT pathway also plays a critical role in tumorigenesis, cancer progression, metastasis, and the development of therapeutic resistance in many human cancers.^42^, ^43^, ^44^, ^45^ Particularly, STAT3 is of interest in cancer biology that can mediate and even promote cancer progression.^46^, ^47^ Interestingly, the JAK–STAT pathway is the downstream signaling of SPP1,^48^, ^49^ and may participate in the SPP1‐mediated tumor progression.

In the current study, we used human ESCA tissue, cell lines, and mouse xenograft models to investigate the role of SPP1 in ESCA progression and especially the development of radiation resistance. We demonstrated that SPP1 could promote ESCA cell progression and facilitate ESCA cell recovery after radiation, which might be one of the mechanisms of radiation resistance. Furthermore, we identified that the JAK–STAT3 pathway participated in the SPP1‐induced radiation resistance.

## 2 MATERIALS AND METHODS

### 2.1 Cell culture and transfection

Eca‐109, Kyse‐150, Kyse‐140, Kyse‐510, and TE‐1 cell lines were obtained from the Cell Bank of the Chinese Academy of Science. Cells were cultured by RPMI‐1640 medium (Gibco, New York, USA) with 10% FBS (Gibco, Brazil). Lentivirus containing SPP1 shRNA, non‐homologous control, SPP1 overexpression sequence, or empty vector were purchased from Shanghai Genechem (SPP1‐targeting oligo sequence: 5′‐ACGAGTCAGCTGGATGACC‐3′; non‐homologous control sequence: 5′‐ATTGCGTTCGCAGTAATCT‐3′).

### 2.2 Cell Counting Kit‐8 (CCK‐8) assays

Cells were planted into 96‐well plates at a density of 3 × 10^3^/well and then incubated at 37°C for 24, 48, 72, 96, and 120 h. Then CCK‐8 reagent (Catalog BA00208, BEIJING BIOSYNTHESIS BIOTECHNOLOGY CO. China) was added, and OD values at 450 nm were detected after 2‐h incubation at 37°C.

### 2.3 EdU incorporation assays

Cells were planted into a 24‐well plate at a density of 1 × 10^5^/well and then incubated at 37°C for 24 h. EdU reagent was added, and cells were fixed with methanol after 2‐h incubation at 37°C. This assay was performed using the Cell‐ Light™ EdU Imaging Detection Kit (Ruibo Biotechnology). Immunofluorescence images were collected by the fluorescence microscope.

### 2.4 Patient sample collection and medical records review

Tissue samples from ESCA patients were collected from the tissue bank of Qilu Hospital. All patients agreed on the research utilization of the collected tissue and all experiments passed ethical review. The tissue microarray (Catalog ESC77, Superbiotek Pharmaceutical Technology Co.) was also used in this study.

### 2.5 Transwell assays

Cells were planted into the upper chamber of 24‐well transwell system plates (Corning, New York, USA). Two hundred microliters of serum‐free medium were added into the upper chamber, 800 μl medium containing 20% FBS (Gibco, Brazil) was added into the lower chambers. Then the migrated cells were fixed with methanol after 24 h‐incubation, stained with 0.1% crystal violet (Beyotime, China), and cell number was counted.

### 2.6 Colony formation assays

Cells were planted into 6‐well plates at a density of 1000 cells/well, and then incubated at 37°C for about 10 days. When visible cell colonies formed, cells were fixed using methanol and stained with 0.1% crystal violet. Then the number of colonies was counted. (A clone must have at least 50 cells.)

### 2.7 Wound healing assays

Cells were planted into 6‐well plates and when cell density reached 70%, a straight line was scratched. Cells were cultured in a serum‐free medium at 37°C for 72 h. The wound photographs were collected at 0, 48, and 72 h.

### 2.8 Western blot

Protein samples were electrophoresed in SDS‐PAGE gels and transferred to PVDF membranes. Then membranes were incubated with the primary antibodies for 12 h at 4°C after blocked for 90 min. Then membranes were incubated with horseradish peroxidase‐labeled secondary antibody (Catalog ZB2301, ZSZB‐BIO, 1:5000) for 1 h and the aim bands were detected. Primary antibodies: anti‐SPP1 antibody (Catalog ab214050, Abcam, 1:3000), anti‐JAK2 antibody (Catalog ab108596, Abcam, 1:3000), anti‐pJAK2 antibody (Catalog ab32101, Abcam, 1:3000), anti‐STAT1 antibody (Catalog ab239968, Abcam, 1:3000), anti‐pSTAT1 antibody (Catalog ab30645, Abcam, 1:3000), anti‐STAT3 antibody (Catalog ab68153, Abcam, 1:3000), anti‐pSTAT3 antibody (Catalog ab76315, Abcam, 1:3000), anti‐pATM antibody (Catalog ab81292, Abcam, 1:3000), anti‐RAD51 antibody (Catalog ab133534, Abcam, 1:3000), anti‐TP53 antibody (Catalog 179,477, Abcam, 1:3000), anti‐p21 antibody (Catalog ab109520, Abcam, 1:3000), and anti‐p27 antibody (Catalog ab32034, Abcam, 1:3000).

### 2.9 Immunofluorescence

Cells were fixed with methanol, blocked with 5% goat serum (ZSGB‐BIO, China), and incubated in PBS with 0.5% Triton X‐100 for 1 h. Then cells were incubated with anti‐γ‐H2AX antibody (Catalog ab81299, Abcam, 1:250), anti‐SPP1 antibody (1:2000), anti‐STAT1 antibody (1:500), anti‐pSTAT1 antibody (1:500), anti‐STAT3 antibody (1:500) and anti‐pSTAT3 antibody (1500) at 4 °C overnight. Then cells were incubated with Alexa Fluor 488 (Catalog A24221, Abbkine Scientific, 1:500) or Dylight 594 (Catalog A24421, Abbkine Scientific, 1:500)‐conjugated secondary antibody for 2 h. Nuclei were stained with DAPI. Images were collected by fluorescence microscope. (Primary antibodies catalog is the same as that of WB).

### 2.10 Immunohistochemistry

The tumor tissue was fixed using formalin, then embedded in paraffin, and sectioned into 4‐mm slides. Immunohistochemistry experiment was performed using the enhanced Polymer Assay kit (PV‐9001; ZSGB ‐ BIO). Then sections were incubated with anti‐SPP1 antibody (1:2000), anti‐STAT3 antibody (1:500), anti‐pSTAT3 antibody (1:500), and anti‐PI3K antibody (Catalog GB111499, Servicebio, 1:800). (Primary antibodies catalog is the same as that of WB).

### 2.11 Irradiation

Irradiation was performed using a linear accelerator (Varian) in Qilu Hospital of Shandong University. Cells were exposed to 6 Gy of radiation the day after inoculation, and mice were irradiated at 6 Gy each time for 3 times with a total of 18Gy.

### 2.12 Flow cytometry

Cells were planted into a 6‐well plate at a density of 3 × 10^5^/well. And cells were exposed to 6 Gy of radiation. Then cells were collected after 12 h and stained with an Annexin‐VFITC/PI Staining Kit (Vazyme Cat. A211‐02). Flowjo software was used for data analysis.

### 2.13 Animal studies

All animal procedures were carried out according to the guidelines of the Association for Assessment and Accreditation of Laboratory Animal Care International. four‐weeks‐old BALB/c male nude mice were used. For tumor growth assays, mice were randomly assigned (*n* = 4 for each group) and *s.c*. inoculated with 2 × 10^7^ ESCA cells. Two animal experiments were conducted in total. In the first experiment, mice were divided into four groups and inoculated with shNC/shSPP1/EV/SPP1 ESCA cells. The mice were cultured for 25 days after inoculation, mainly to detect the effect of SPP1 itself on the tumor‐forming ability of ESCA cells in vivo. The second animal experiment was to divide the mice into six groups (0GY + shNC + DMSO, 9GY + shNC + DMSO, 0GY + shNC+SH‐4‐54, 9GY + shNC + SH‐4‐54, 0GY + shSPP1 + DMSO, 9GY + shSPP1 + DMSO). Administration of 10 mg/kg intraperitoneal injection was initiated 7 days after inoculation. The irradiation of animals was performed using the radiation apparatus in Qilu Hospital, with a single dose of 6GY and a total of 18GY. Mice were cultured for a total of 30 days. Then mice were sacrificed, and the tumor tissues were collected. Tumor volumes were calculated as (length × width^2^)/2.

### 2.14 Human Apoptosis Array Kit

Cells were inoculated into a 6‐well plate at a density of 3 × 10^5^/well, and exposed to 6 Gy of radiation, then cells were collected 12 h later. The apoptosis data was assessed by the Human Apoptosis Array Kit (Catalog Number ARY009) following the manufacturer's protocol.

### 2.15 Bioinformatics

The RNA‐seq datasets (GSE5364, GSE20347, GSE23400, GSE38129, GSE77861) were downloaded from the Gene Expression Omnibus (GEO) repository (https://www.ncbi.nlm.nih.gov/geo/) and the differentially expressed genes (DEGs) were calculated by the GEO2R (https://www.ncbi.nlm.nih.gov/geo/info/geo2r.html) tool. DEGs were defined as |log2FoldChange| > 2 and adjusted *p*‐value <0.05. RNAseq data of human ESCA tissue were downloaded from the TCGA database using the GDC Data Portal (https://gdc‐portal.nci.nih.gov/) and the metadata was further retrieved by *R* (https://www.r‐project.org/). One‐way ANOVA was used for statistical analysis. The mRNA expression data of TCGA‐ESCA included a total of 171 tumor samples consisting of 160 tumor samples and 11 normal samples. The sequencing data were all publicly available and no ethical issues were involved. The disease‐free survival analysis between SPP1^high^ and SPP1^low^ ESCA patients in the TCGA dataset was conducted by an online tool (http://gepia.cancer‐pku.cn/).

### 2.16 Statistics

Statistical Package for Social Science (SPSS) and GraphPad Prism 7.0 were used for our statistical analysis. One‐way ANOVA, Pearson correlation tests, or two‐tailed student's *t*‐tests were used to analyze data, and Kaplan–Meier survival data were analyzed with the log‐rank test. Each experiment was repeated three times. The sample size was chosen without a predetermined effect size. Data were presented as the mean ± standard error of the mean (SEM) or the mean ± standard deviation (SD). A *p*‐value <0.05 was considered statistically significant.

## 3 RESULTS

### 3.1 Overexpression of SPP1 associated with poor prognosis in ESCA

**FIGURE 1:**
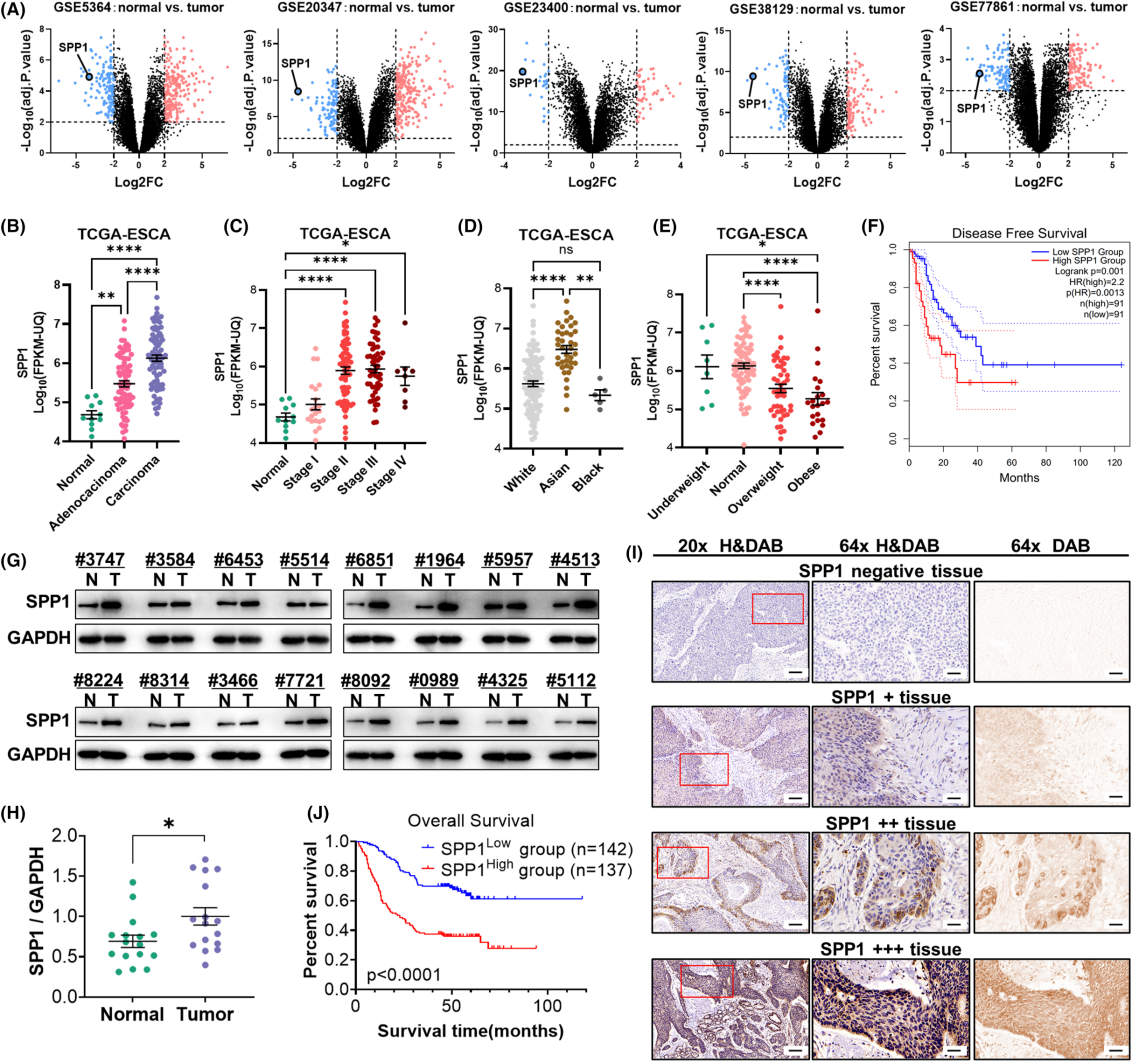
Overexpression of SPP1 is associated with poor prognosis in ESCA. (A) Multiple RNA‐seq datasets cross‐validated and showed the upregulated SPP1 expression in ESCA compared to normal tissue. (B) Both esophageal carcinoma and adenocarcinoma had higher SPP1 expression compared to normal tissue and the esophageal carcinoma had the most upregulated SPP1 expression indicated by the TCGA dataset. (C) SPP1 expression positively correlated with ESCA tumor stages. (D) Asian ESCA patients had significantly higher SPP1 expression compared to other races. (E) Overweight and obese ESCA patients had more SPP1 expression. (F) The disease‐free survival was significantly lower in patients with high Spp1 expression indicated by the TCGA dataset. (G and H) The higher SPP1 protein expression in ESCA tissue was validated in patient samples collected in our institution. (Sample named using the last four numbers of patients' hospital number) (I) Representative IHC images of SPP1 in ESCA patient samples with intensities −, +, ++, and +++. (J) Kaplan–Meier curves of overall survival data in our patient cohort with SPP1 staining. ESCA of patients with high SPP1 protein expression (++/+++) had significantly lower Overall Survival according to follow‐up records in our institution. *p < 0.05, **p < 0.01, ***p < 0.001, ****p < 0.0001. 20X image scale bar = 100 μm, 63X image scale bar = 20 μm.

To identify the specific role of SPP1 in ESCA, we first analyzed its prognostic values. We calculated the differentially expressed genes (DEGs) of multiple human ESCA RNAseq datasets (GSE5364, GSE20347, GSE23400, GSE38129, GSE77861) from the Gene Expression Omnibus (GEO) database repository and found that compared to the normal tissue, the ESCA tissue had a significantly higher SPP1 gene expression (Figure 1A). In addition, we analyzed information from The Cancer Genome Atlas (TCGA) dataset, and the results showed that both the ESCA carcinoma and adenocarcinoma tissue had a higher SPP1 expression compared to normal tissue, while the carcinoma tissue had the highest SPP1 expression (Figure 1B). The SPP1 expression is positively correlated with the ESCA tumor stage (Figure 1C). Interestingly, Asian ESCA patients had the highest SPP1 expression compared to other races (Figure 1D), and the overweight or obese ESCA patients had a lower SPP1 gene expression compared to normal weight or underweight patients (Figure 1E). Further exploration of the TCGA database showed that ESCA patients with a higher SPP1 gene expression had a significantly lower disease‐free survival (Figure 1F). Given that the RNAseq results could only indicate the RNA level but not the protein level of SPP1 gene expression, we retrieved the ESCA patient tissue from the cancer tissue bank in our institution and analyzed the SPP1 protein expression by western blotting. Consistent with the RNAseq results, ESCA tissue had more SPP1 protein expression than the normal tissue (Figure 1G,H). To further explore the prognostic role of SPP1 in ESCA, we undertook immunohistochemical staining of 279 patients from our ESCA cohort and classified these patients into 2 groups based on the SPP1 expression level (SPP1^high^ and SPP1^low^) (Figure 1I). We compared the Overall Survival between groups according to their follow‐up medical records, and the result showed that high SPP1‐expressing ESCA patients had a significantly lower overall survival (Figure 1J). The results that overexpression of SPP1 associated with poor prognosis in ESCA strongly indicated a critical role of SPP1 in ESCA progression.

### 3.2 Knockdown of SPP1 in human ESCA cells enhanced radiation sensitivity

**FIGURE 2:**
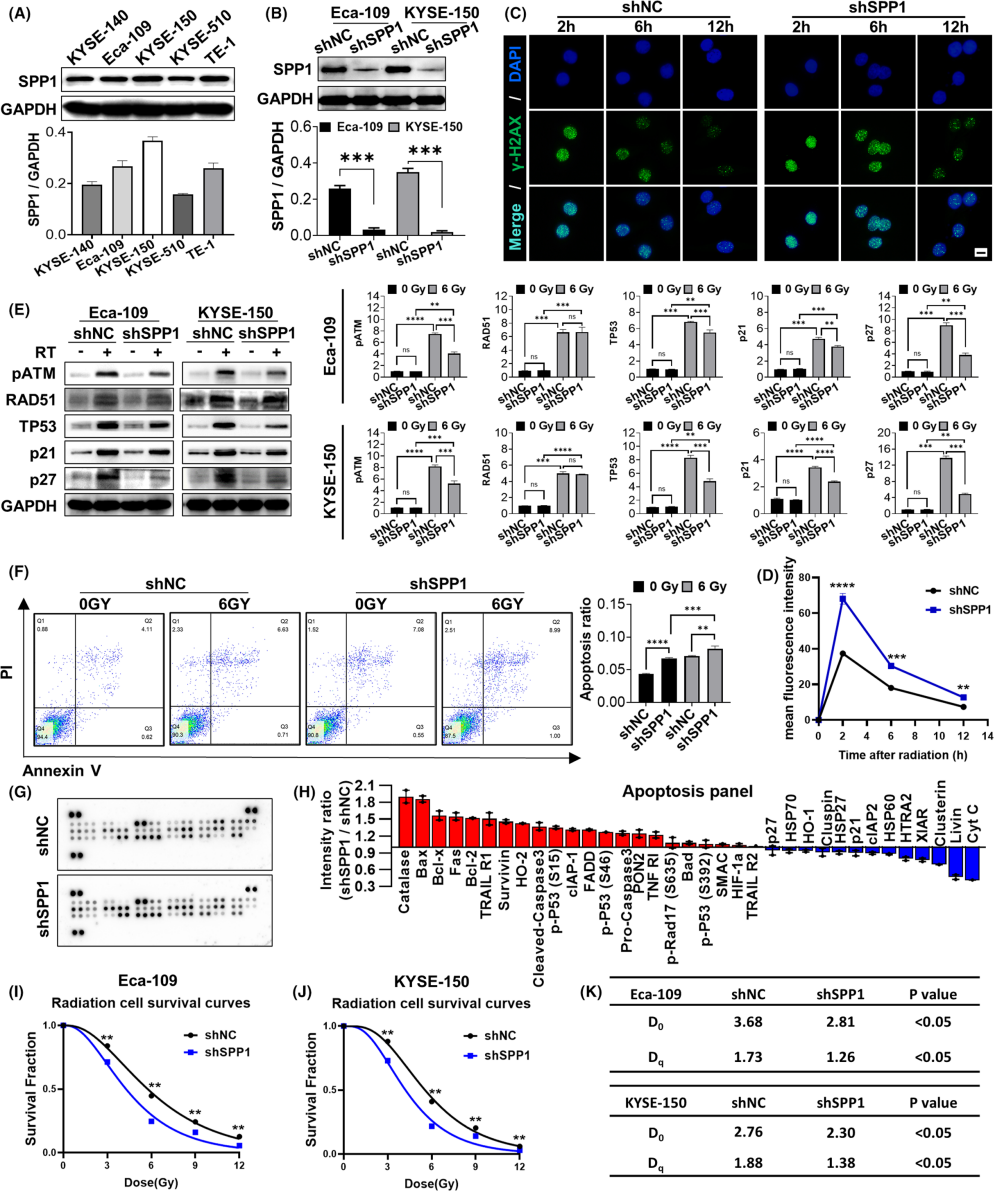
Knockdown of SPP1 in human ESCA cells enhanced radiation sensitivity. (A) Protein levels of SPP1 in KYSE‐140, Eca‐109, KYSE‐105, KYSE‐510, TE‐1 cell lines. (B) Protein levels of SPP1 in SPP1‐knockdown and control ESCA cells (Eca‐109 and KYSE‐150). (C and D) Dynamic levels of γ‐H2AX in SPP1‐knockdown and control Eca‐109 cells 2, 6, and 12 h post‐radiation. Scale bar = 50 μm (E) Relative expression of pATM, RAD51, TP53, p21, and p27 in SPP1‐knockdown and control ESCA cells (Eca‐109 and KYSE‐150), 2 h post‐radiation. (F) Apoptosis assays of SPP1‐knockdown and control KYSE‐150 cells with or without radiation. (G and H) SPP1 knockdown altered the apoptosis‐related protein profile indicated by the chip array. (I–K) Survival curves of SPP1‐knockdown and control ESCA cells (Eca‐109 and KYSE‐150). D_0_ and D_q_ values were calculated fitting to a multi‐target model. *p < 0.05, **p < 0.01, ***p < 0.001, ****p < 0.0001. shNC, negative control; shSPP1, SPP1‐knockdown.

To study the molecular mechanisms of SPP1 in ESCA progression and radiation resistance, we first checked the SPP1 expression levels in five human ESCA cell lines, which all showed abundant SPP1 expression. The human ESCA cell lines Eca‐109 and KYSE‐150 were further chosen for subsequent experiments because of their relatively higher SPP1 expression (Figure 2A). Then we constructed the short‐hairpin RNA targeting the SPP1 gene (shSPP1) or negative control (shNC) and utilized the lentivirus to generate the stable SPP1‐knockdown Eca‐109 and KYSE‐150 cell lines (Figure 2B). Despite the multiple mechanisms involved in irradiation‐mediated cell death, DNA damage is still the principal reason, while the impaired double‐strand break (DSB) repair is a major cause of radio resistance.^50^ To explore the relationship between SPP1 and DSB repair, we examined the dynamic levels of γ‐H2AX, a hallmark of DSB, after radiation. We found that SPP1 knockdown significantly increased the γ‐H2AX signals within 12 h after radiation (results of KYSE‐150 cells shown in Figure 2C,D, results of ECA‐109 cells shown in Figure S1A,B), indicating that SPP1 knockdown impeded DSB repair post‐radiation. Now that we found that SPP1 knockdown could reduce the repairability after DNA damage in ESCA cells, then we added recombinant human SPP1(0.5 μg/mL) to SPP1‐knockdown cell lines and found that the reduced repair ability after DNA damage could be reversed (Figure S1H). We also detected other proteins related to DNA repair 2 h post‐radiation, and found that the expressions of pATM, TP53, P21, and P27 in the SPP1 knockdown cells were all decreased while no significant changes were seen in RAD51 and PARP1, suggesting that SPP1 knockdown may reduce the ability of ESCA cells to stop the cell cycle for DNA repair after DNA damage (Figure 2E, Figure S2B). Then we tested the irradiation‐induced apoptosis, which is a primary way of tumor cell death post‐radiation.^51^ Flow cytometry showed that the ratio of PI^+^AnnexinV^+^ apoptotic tumor cells increased in the SPP1 knockdown group compared with the NC group, and further increased after radiation (results of Eca‐109 cells shown in Figure 2F, results of KYSE‐150 cells shown in Figure S1C). To perform a more comprehensive analysis of the changes in apoptosis‐related protein, we used a human apoptosis array kit to compare the SPP1 knockdown group to the NC group. The results showed an increase in pro‐apoptotic proteins such as Bax, cleaved‐caspase3, FADD, and a decrease in anti‐apoptotic proteins such as Livin and XIAP in the SPP1 knockdown group (Figure 2G,H). Survival curves of SPP1‐knockdown and control ESCA cells (Eca‐109 and KYSE‐150 cell lines) post‐radiation were then analyzed using a single‐hit multi‐target model (Figure 2I,J). The *D*_0_(*D*_q_) values of the shSPP1 group in Eca‐109 and KYSE‐150 were 2.81(1.26) and 2.30(1.38), respectively, which were significantly lower than that in the shNC group 3.68(1.73) and 2.76(1.88), respectively (Figure 2K). All these results suggested that downregulation of SPP1 could impede DNA damage repair ability in human ESCA cells post‐radiation and lead to more prominent tumor apoptosis. Thus, SPP1‐targeting therapy could enhance radiation sensitivity in ESCA.

### 3.3 SPP1 knockdown impeded tumor progression in human ESCA cells synergistically with radiation

**FIGURE 3:**
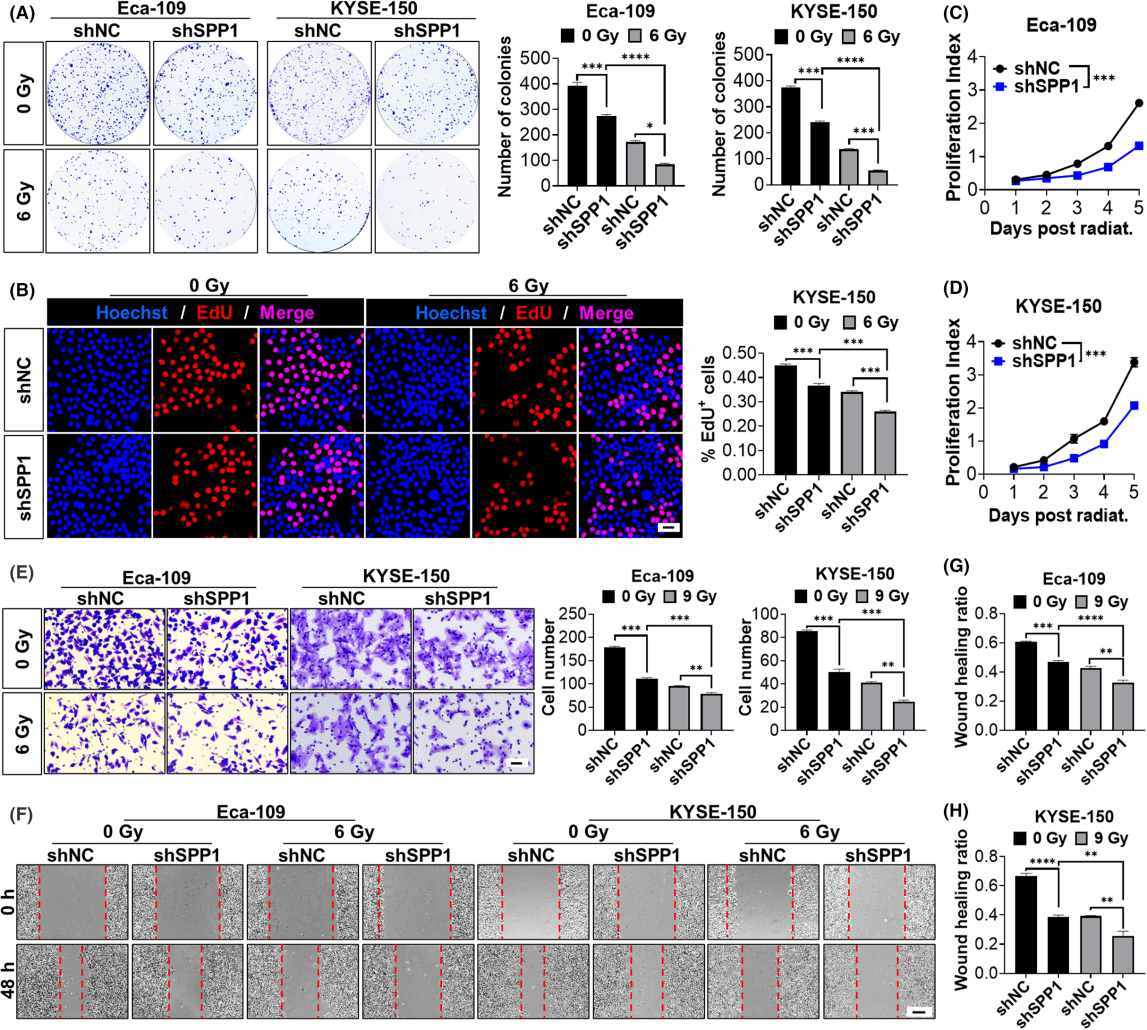
SPP1 knockdown impeded tumor progression in human ESCA cells with or without radiation. SPP1 knockdown impeded ESCA cells' colony formation ability (A) and cell proliferation (B) synergistically with radiation, Scale bar = 100 μm. (C and D) Proliferation curves of SPP1‐knockdown and control cells. SPP1 knockdown further decreased the migration ability (E) (Scale bar = 100 μm) and wound healing rate (F–H) (Scale bar = 400 μm) of the ESCA cells survived the radiation. *p < 0.05, **p < 0.01, ***p < 0.001, ****p < 0.0001. shNC, negative control; shSPP1, SPP1‐knockdown.

Previous studies suggested SPP1 itself can affect the proliferation and migration of tumor cells in lung cancer and colorectal cancer,^52^, ^53^ however, its role in ESCA is less studied. In addition, no previous studies have focused on the role of SPP1 post‐radiation. To address this question, we conducted a series of experiments using Eca‐109 and KYSE‐150 human ESCA cell lines. The colony formation assays showed that the total numbers of colonies in both cell lines were decreased in the SPP1 knockdown group without radiation and were further decreased in the SPP1 knockdown group after radiation (Figure 3A). The results of the EdU incorporation experiment (results of KYSE cells shown in Figure 3B, results of Eca‐109 cells shown in Figure S1D) and CCK‐8 assay (Figure 3C,D) showed that the SPP1 knockdown cells had a lower proliferation index without radiation, and more dramatically after radiation. Besides cell proliferation, we also assessed the migration ability after SPP1 knockdown with or without radiation. The Transwell assay (Figure 3E) and Wound Healing assay (Figure 3F–H) showed a decreased migration rate in the SPP1 knockdown group compared to the NC group before radiation while the migration rate further decreased after radiation. All these results confirmed that SPP1‐targeting therapy itself could impede ESCA cell proliferation and migration ability. More importantly, the combination of SPP1‐targeting therapy and the standard clinical ESCA treatment, radiation, yielded more promising outcomes for ESCA treatment.

### 3.4 Radiation increased SPP1 expression and overexpression of SPP1 promoted radiation resistance of human ESCA cells

**FIGURE 4:**
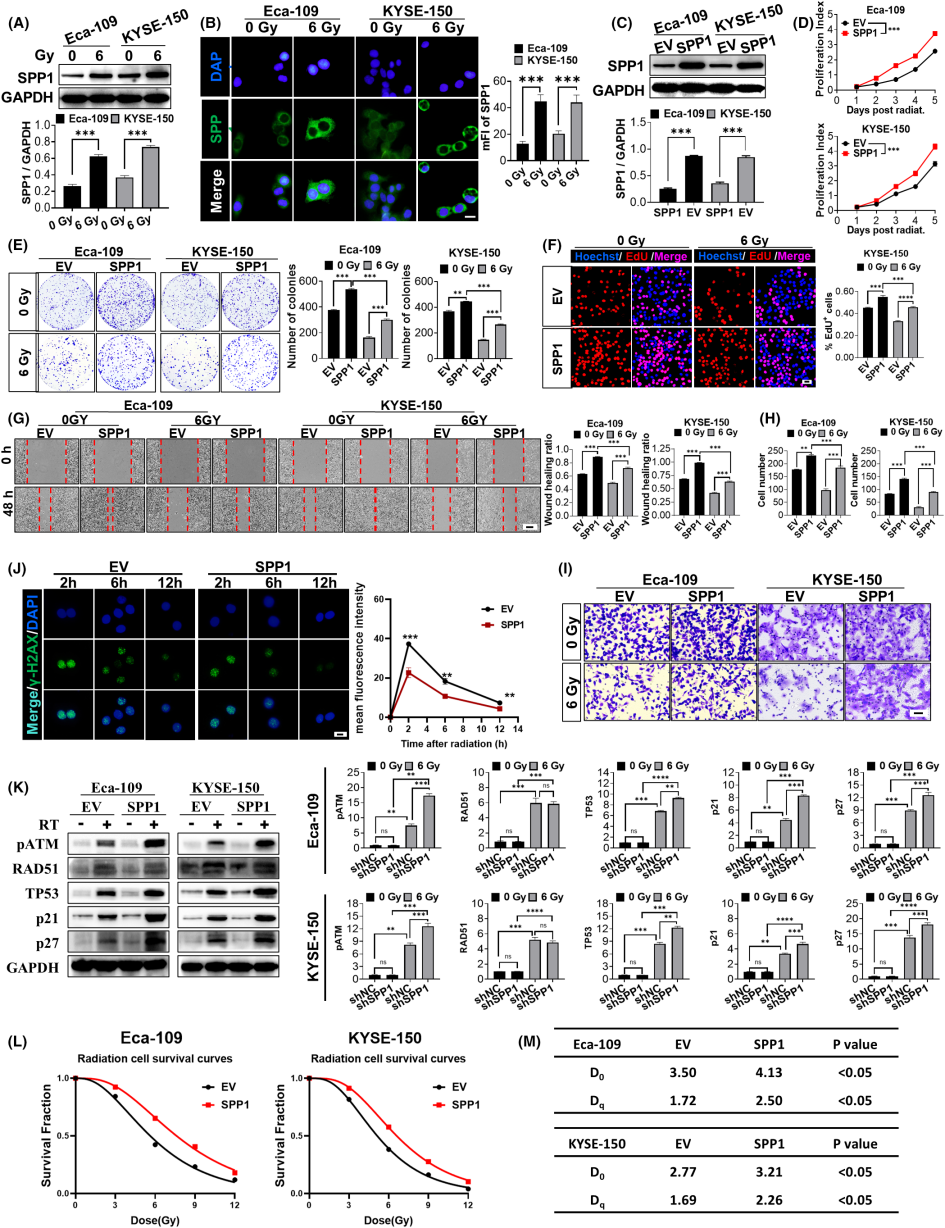
Radiation increased SPP1 expression and overexpression of SPP1 promoted radiation resistance of human ESCA cells. Protein levels of SPP1 increased after radiation in western blotting (A) and cell immunofluorescence, Scale bar = 50 μm. (B). (C) Protein levels of SPP1 in SPP1‐overexpression and control ESCA cells (Eca‐109 and KYSE‐150 cell lines). (D) Proliferation curves of SPP1‐overexpressing and control cells. SPP1 overexpression strengthened ESCA cells' colony formation ability (E) and cell proliferation (F), especially the ESCA cells that survived radiation, scale bar = 100 μm. SPP1 overexpression increased the wound healing rate, Scale bar = 400 μm (G) and migration ability, Scale bar = 100 μm (H‐I) of the ESCA cells survived radiation. (J) IF staining of γH2AX in SPP1‐overexpressing and control Eca‐109 cells, 2, 6, and 12 h post‐radiation. Scale bar = 50 μm (K) Relative expression of pATM, RAD51, TP53, p21, and p27 in SPP1‐overexpression and control ESCA cells (Eca‐109 and KYSE‐150), 2 h post‐radiation. (L and M) Survival curves of SPP1‐overexpressing and control ESCA cells (Eca‐109 and KYSE‐150). *D*_0_ and *D*_q_ values were calculated fitting to a multi‐target model. *p < 0.05, **p < 0.01, ***p < 0.001, ****p < 0.0001. EV, empty vector; SPP1, lentivirus containing SPP1.

Since we had demonstrated the effect of SPP1 downregulation on the proliferation, migration, and radiation sensitivity of ESCA cells, we wondered whether those cells would normally change SPP1 expression after radiation, which is the standard clinical treatment for ESCA patients.^8^ Thus, we tested the protein level of SPP1 after radiation and interestingly found that ESCA cells increase SPP1 expression (Figure 4A,B), suggesting that ESCA cells could utilize SPP1 to promote their recovery from radiation and develop radio‐resistance. To demonstrate this hypothesis, we constructed SPP1‐overexpressing ESCA cell lines (SPP1 group) and empty vector control ESCA cell lines (EV group) using lentivirus (Figure 4C). As expected, the results of the CCK‐8 assay showed that the overexpression of SPP1 increased ESCA cells' proliferation (Figure 4D). And the SPP1 group showed higher colony formation rates than the EV group with or without radiation (Figure 4E). In addition, the EdU incorporation assay showed that overexpression of SPP1 stimulated ESCA cell division, especially after radiation, which suggested that ESCA cells with more SPP1 expression were going through more active proliferation post‐radiation (results of KYSE cells shown in Figure 4F, results of Eca‐109 cells shown in Figure S1G). Then we assessed the migration ability of the SPP1 and EV group by the transwell assay (Figure 4H–I) and Wound Healing assay (Figure 4G). The results showed that the SPP1‐overexpressing group had an increased migration rate and a faster wound healing rate, especially in the ESCA cells that survived radiation. These results suggested that the inhibitory effect of radiation on the proliferation and migration of ESCA cells was counteracted by the increased SPP1 expression. As we had demonstrated that SPP1 knockdown impeded DNA damage repair in ESCA cells, we assumed that the phenomenon we just uncovered here, the increased SPP1 expression in ESCA cells after radiation, was a novel mechanism for ESCA to enhance its radiation resistance by promoting DNA damage repair. To examine our hypothesis, we examined the level of γ‐H2AX 6 h post‐radiation. Results showed that overexpression of SPP1 significantly decreased the γ‐H2AX signals (results of Eca‐109 cells shown in Figure 4J, results of KYSE‐150 cells shown in Figure S1E‐F) compared to the EV group, which indicated that SPP1 upregulation could promote DSB repair post‐radiation. We also found the expressions of pATM, TP53, P21, and P27 in SPP1‐overexpression cells were increased while no significant changes were seen in RAD51 and PARP1, 2 h post‐radiation, which is just as we suspected, suggesting that SPP1 overexpression can increase the ability of ESCA cells to stop the cell cycle for DNA repair after DNA damage (Figure 4K, Figure S2A). Survival curves of SPP1‐overexpressing and control ESCA cells post‐radiation were then analyzed using a single‐hit multi‐target model (Figure 4L). The *D*_0_(*D*_q_) values of the SPP1 group in Eca‐109 and KYSE‐150 were 4.13 (2.50) and 3.21(2.26), higher than that in the EV group (3.50(1.72) and 2.77(1.74), respectively) (Figure 4M). These results suggested that upregulation of SPP1 could promote DNA damage repair, decrease tumor cell death ratio, and increase radiation resistance in ESCA cells. Given the novel phenomenon that the human ESCA cells increased SPP1 expression after radiation, we propose that SPP1 significantly contributes to the tumor relapse and the development of therapeutic resistance to radiotherapy in human ESCA.

### 3.5 SPP1 promoted the therapeutic resistance to radiation through the JAK2/STAT3 pathway

**FIGURE 5:**
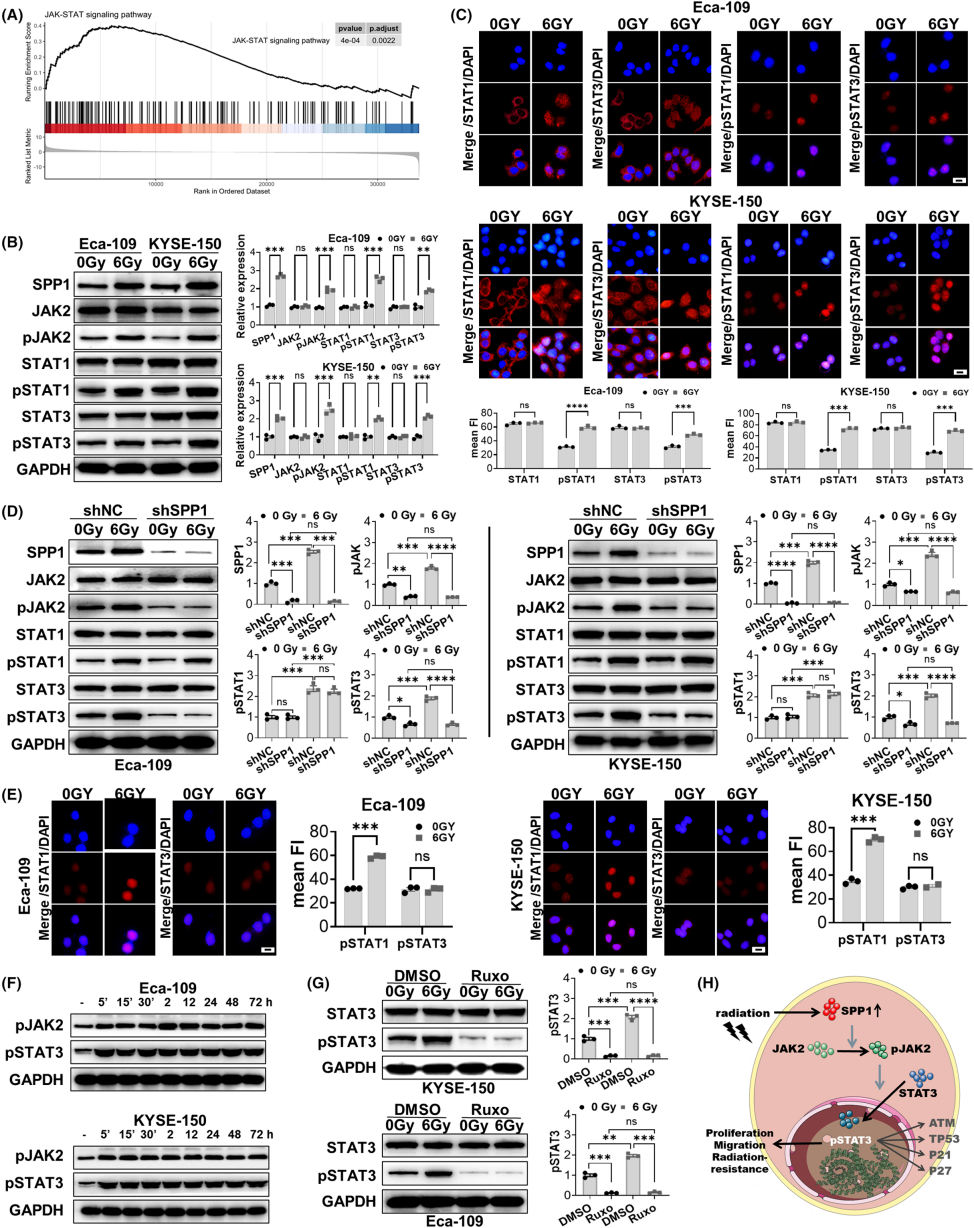
SPP1 promoted the therapeutic resistance to radiation through the JAK2/STAT3 pathway. (A) Gene Set Enrichment Analysis (GSEA) results of the JAK–STAT signaling pathway. Protein levels of JAK2, STAT1, STAT3, and their phosphorylation levels, together with SPP1, after radiation in human ESCA cells (Eca‐109 and KYSE‐150 cell lines) in western blotting (B) and cell immunofluorescence (C). Protein levels of JAK2, STAT1, STAT3, and their phosphorylation levels after SPP1 knockdown in western blotting (D) and cell immunofluorescence (E). (F) Western blot results of pJAK2 and pSTAT3 with protein extracted at different time points after radiation in ESCA cells (Eca‐109 and KYSE‐150 cell lines). (G) Protein levels of phosphorylated STAT3, 12 h post‐radiation, with the administration of Ruxolitinib (Ruxo, 2.8 nM, dissolved in DMSO). (H) Model of the SPP1/JAK2/STAT3 pathway in tumor progression and radio‐resistance in ESCA. *p < 0.05, **p < 0.01, ***p < 0.001, ****p < 0.0001. Scale bar = 50 μm.

To further explore the specific molecular mechanisms of SPP1 in ESCA progression after radiation, we decided to investigate the downstream pathway of SPP1. A GSEA (Gene Set Enrichment Analysis) was conducted between low and high SPP1 expression data using the TCGA database, and we found that the JAK–STAT signaling pathway had significant enrichment with SPP1 high‐expression phenotype (Figure 5A). The JAK–STAT signaling pathway is reported to be implicated in human cancer development, progression, metastasis, and resistance to treatment.^42^ Previous studies have confirmed that the JAK–STAT pathway is related to radiation resistance in colorectal cancer and prostate cancer,^43^, ^44^, ^45^ while STAT‐inhibitors can increase radiosensitivity and improve the therapeutic effect of radiation.^44^, ^45^ Thus, we would like to know if the JAK–STAT pathway got activated in the human ESCA cells after radiation. We checked the expression of JAK2, STAT1, STAT3, and the phosphorylation state of these proteins post‐radiation, and surprisingly found that the total expression level of JAK2, STAT1, and STAT3 did not change while their phosphorylation level increased, together with SPP1 expression increase 12 h post‐radiation (Figure 5B,C). And the immunofluorescence results also showed that STAT1 and STAT3 were partially transferred from cytoplasm to nucleus after radiation (Figure 5C), suggesting the phosphorylation activation of STAT1 and STAT3. Interestingly, the JAK–STAT pathway was previously reported to be downstream signaling of SPP1 in murine mammary epithelial tumor and breast cancer,^48^, ^49^ and our GSEA results also showed that the JAK–STAT signaling pathway had significant enrichment with SPP1 high‐expression phenotype. Thus, we hypothesized that the post‐radiation elevated expression of SPP1 promoted human ESCA cell recovery and contributed to the radiation resistance through the JAK/STAT pathway. To address our hypothesis, we compared the phosphorylation level of JAK2, STAT1, and STAT3 between shSPP1 and shNC groups with or without radiation. As expected, we found a novel phenomenon that the level of pJAK2 and pSTAT3 was decreased after SPP1 knockdown without radiation, then more important phenomenon was that the post‐radiation increase of pJAK2 and pSTAT3 was restrained in the shSPP1 group compared to shNC group, while the pSTAT1 with no difference (Figure 5D,E). These results indicated that SPP1 specifically activated the JAK2‐STAT3 pathway post‐radiation while the elevated pSTAT1 was not related to SPP1. To verify that SPP1 directly induced the activation of the JAK2‐STAT3 pathway, we added recombinant human SPP1 into the culture medium of SPP1‐knockdown cells, and we detected the relative expression levels of pJAK2 and pSTAT3 at different time points. We surprisingly found that SPP1 could rapidly activate the JAK2‐STAT3 pathway within 5 min, and this effect may last for at least 72 h (Figure 5F). To further confirm that the phosphorylation of STAT3 was indeed due to JAK2 activation, we treated ESCA cells with the JAK2 inhibitor Ruxolitinib (Ruxo) with or without radiation and analyzed the phosphorylation level of STAT3. We found that the phosphorylation levels of STAT3 were significantly inhibited after JAK2 inhibition both with and without radiation, indicating that STAT3 was regulated by JAK2 (Figure 5G). We also made an illustration of the suggested pathway (Figure 5H).

### 3.6 Pharmacological inhibition of the JAK2‐STAT3 pathway abolished SPP1‐mediated therapeutic resistance to the radiation

**FIGURE 6:**
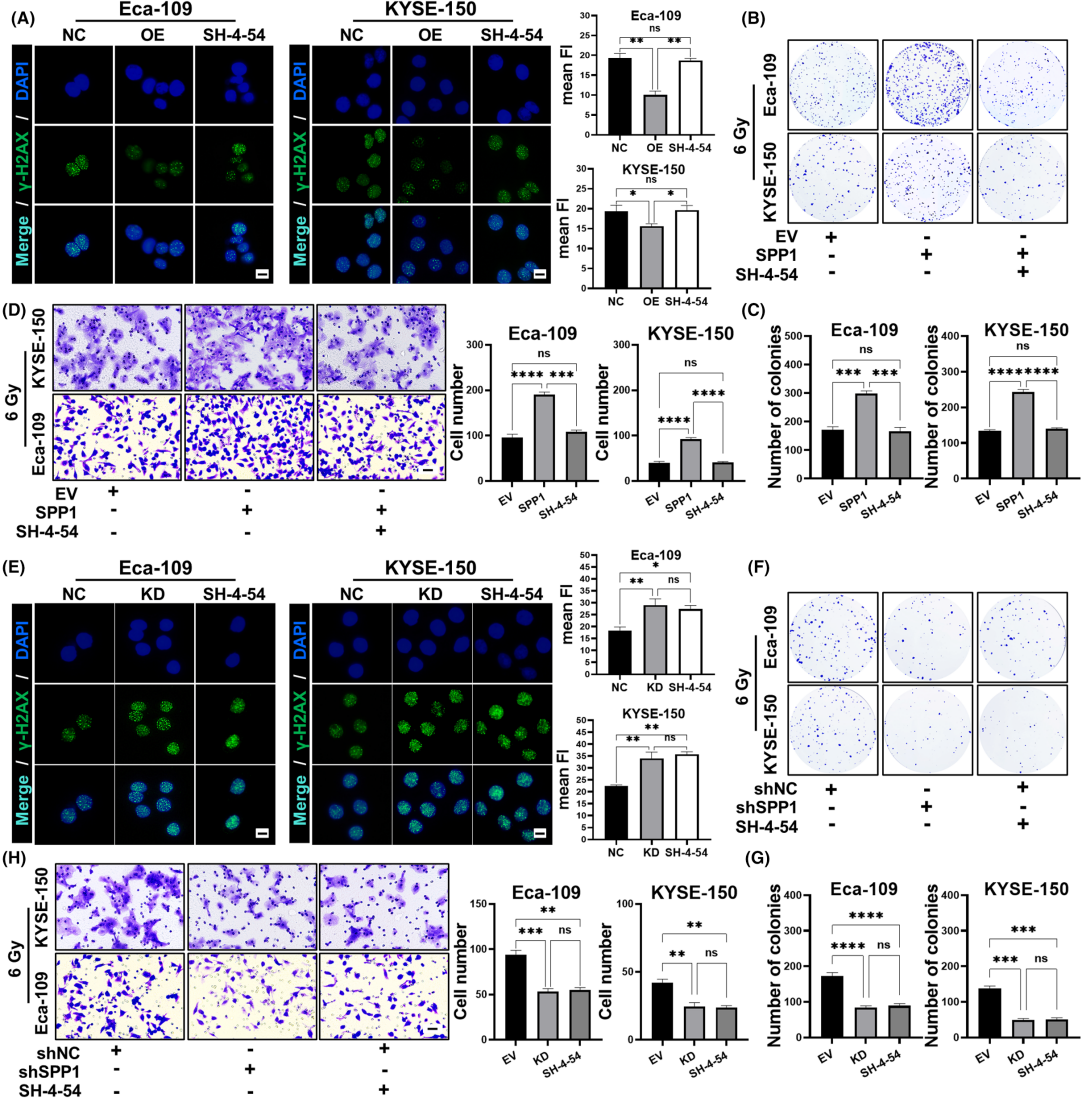
Pharmacological inhibition of the JAK/STAT3 pathway abolished SPP1‐mediated therapeutic resistance to the radiation. IF staining of γH2AX 6 h post‐radiation (Scale bar = 50 μm) (A), colony formation assays (B and C), and transwell assays (Scale bar = 100 μm) (D) in ESCA cells (Eca‐109 and KYSE‐150 cell lines) post‐radiation, of three groups: EV group (empty vector), SPP1 group (SPP1 overexpression), and SH‐4‐54 group (SPP1 overexpression + SH‐4‐54 [inhibitor of STAT3, 300 nM, dissolved in DMSO]). IF staining of γH2AX 6 h post‐radiation (Scale bar = 50 μm) (E), colony formation assays (F and G), and transwell assays (Scale bar = 100 μm) (H) in ESCA cells (Eca‐109 and KYSE‐150 cell lines) of three group: shNC group (negative control), SPP1 group (SPP1 knockdown), and SH‐4‐54 group (SH‐4‐54 [inhibitor of STAT3, 300 nM, dissolved in DMSO]). *p < 0.05, **p < 0.01, ***p < 0.001, ****p < 0.0001.

After delineating the relationship between SPP1 and the downstream signal JAK2‐STAT3, we wanted to know whether blocking the JAK2‐STAT3 pathway could eliminate the radio‐resistance phenomenon induced by SPP1 overexpression. Thus, we added the STAT3 phosphorylation inhibitor SH‐4‐54 (300 nM, dissolved in DMSO) in the SPP1 overexpression group post‐radiation. So, there were three groups in the experiment: EV group (empty vector + DMSO) as negative control; SPP1 group (SPP1 overexpression + DMSO) to study the pure radio‐resistance effect of SPP1; SH‐4‐54 group (SPP1 overexpression + SH‐4‐54) to study how STAT3 blockade affected the radio‐resistance induced by SPP1‐overexpression. We examined the levels of γ‐H2AX 6 h post‐radiation, and as expected, SPP1‐overexpression significantly decreased the γ‐H2AX signals, and when the activation of STAT3 was blocked, the signal reduction of γ‐H2AX was almost eliminated (Figure 6A). Colony formation assays (Figure 6B,C) and transwell assays (Figure 6D) showed that SPP1‐overexpression could increase the post‐radiation colony formation and migration ability of ESCA cells, and these increases could be inhibited by blocking STAT3. These results suggested that inhibition of STAT3 could abolish the radio‐resistance effect induced by SPP1 upregulation. Therefore, we demonstrated that SPP1 upregulation induced radiation resistance through the downstream STAT3 activation. To more fully confirm that SPP1 affects radiosensitivity through the downstream JAK–STAT pathway, we wanted to investigate the effects of SPP1 knockdown and STAT activation inhibition on radiosensitivity, respectively. Thus, 3 experimental groups were used in the following studies: shNC group (shNC + DMSO); shSPP1 group (SPP1‐knockdown + DMSO); SH‐4‐54 group (shNC + SH‐4‐54). We first detected the levels of γ‐H2AX 6 h post‐radiation, as expected, both SPP1 knockdown and inhibition of STAT3 could increase the γ‐H2AX signals (Figure 6E). Then colony formation assays (Figure 6F,G) and transwell assays (Figure 6H) showed that both SPP1 knockdown and inhibition of STAT3 could decrease the wound healing rate and proliferation ability of ESCA cells post‐radiation. All these results suggested that the JAK2‐STAT3 pathway was a major downstream pathway of SPP1, and both its inhibition and SPP1 knockdown could enhance the therapeutic effect of radiation on ESCA, which may be potential clinical therapeutic targets of ESCA.

### 3.7 SPP1 knockdown and STAT3 inhibition promoted the curative effect of radiation on ESCA in vivo

**FIGURE 7:**
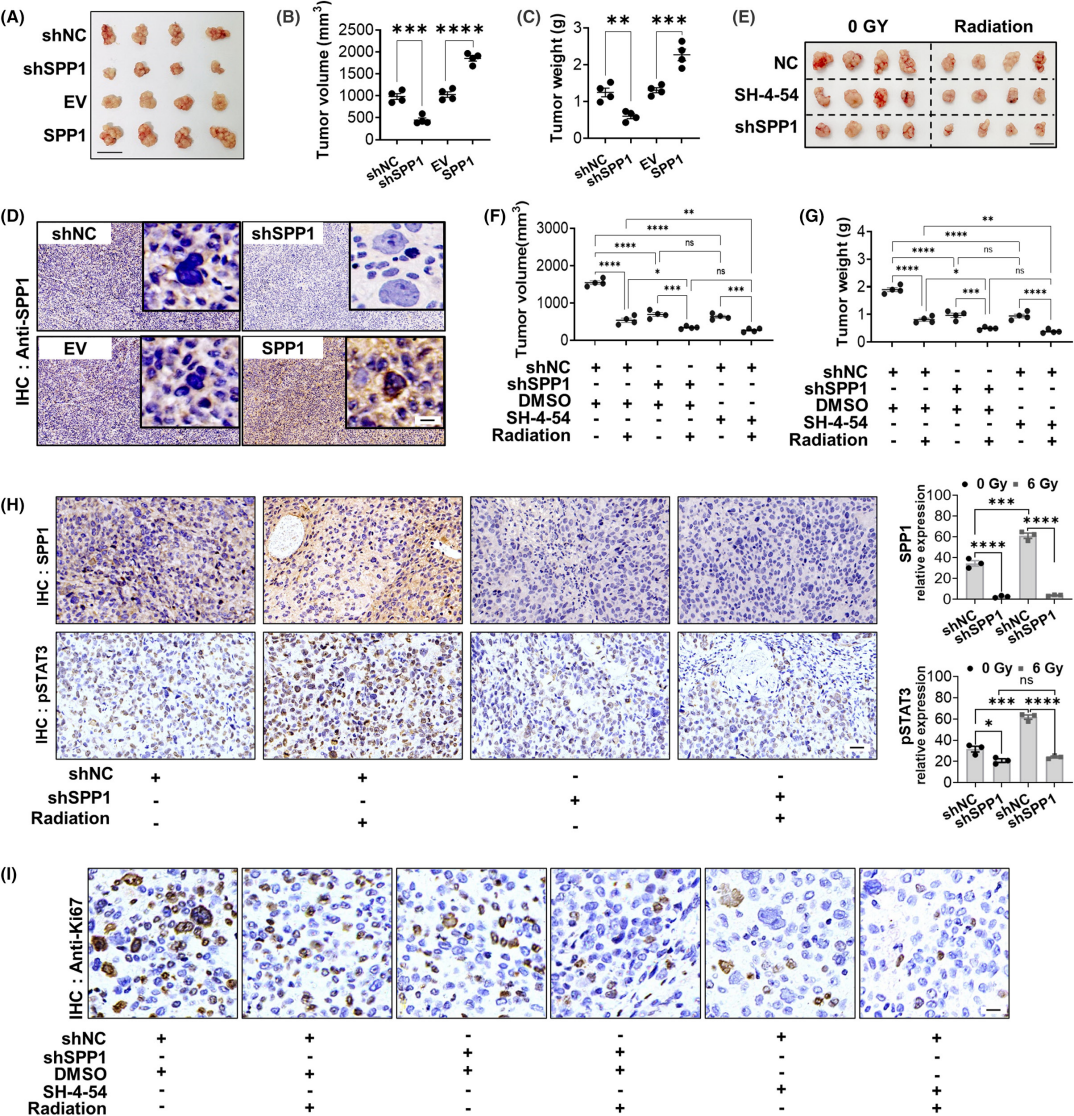
Both SPP1 genetic knockdown and pharmacological inhibition of the JAK2/STAT3 pathway can strengthen radiation sensitivity in a mice ESCA xenograft model. (A–C) ESCA xenograft with SPP1 knockdown tumor cells had the smallest tumor volume and weight while ESCA xenograft with SPP1 overexpression had the largest tumor volume and weight. (D) Representative IHC images of xenograft tumor tissue histology with SPP1 expression. Scale bar = 50 μm. (E and G) Volume and weight of xenografts with or without SH‐4‐54, shSPP1, and radiation. (H) The immunohistochemical staining of SPP1 and pSTAT3 with or without radiation. Scale bar = 200 μm. (I) Ki67 decreased in both the SH‐4‐54 group and the SPP1‐knockdown group compared to the NC group. Scale bar = 100 μm. *p < 0.05, **p < 0.01, ***p < 0.001, ****p < 0.0001.

So far, all the forementioned experiments were done in vitro, which might not closely mimic the real tumor environment in vivo. Thus, we would like to conduct in vivo experiments to further validate our results. We constructed a tumor xenograft model in nude mice to evaluate the role of SPP1 in ESCA tumor progression and resistance to radiation. In the first experiment, 16 nude mice were randomly divided into 4 groups and received subcutaneous injection of shNC, shSPP1, EV, or SPP1‐overexpression Eca‐109 cells, then the mice tumors were irradiated at a dose of 6GY three times for a total of 18GY. Twenty‐five days after radiation, tumors were collected (Figure 7A). We measured the tumor volume and tumor weight in each group and the results showed that the SPP1 overexpression group had the largest tumor volume and weight while the SPP1 knockdown group had the smallest (Figure 7B,C). The overexpression or knockdown of SPP1 was verified in the corresponding groups by immunohistochemistry staining (Figure 7D). This experiment confirmed the favorable effects of SPP1 on ESCA progression. In the second experiment, 24 nude mice were randomly divided into 6 groups: NC group (shNC + DMSO); shSPP1 group (shSPP1 + DMSO); SH‐4‐54 group (shNC + SH‐4‐54) and their corresponding irradiated groups (Figure 7E). We found that both giving STAT3 inhibitor or SPP1‐knockdown could decrease the tumor volume and tumor weight compared to the negative control group, which was more obvious after radiation (Figure 6E–G). This was consistent with the in vitro experiment. Next, we checked the expression of SPP1 and pSTAT3 in the NC group, shSPP1 group, and their corresponding irradiated groups by immunohistochemistry. The results were consistent with previous cell experiments that the expression of SPP1 and pSTAT3 increased after radiation, while the post‐radiation increase of STAT3 was suppressed when SPP1 was knocked down, indicating that radiation could increase pSTAT3 by increasing the expression of SPP1 (Figure 7H). We also checked the expression level of Ki67, which is an indicator of cell proliferation, and as expected, we found the expression of Ki67 decreased in both the STAT‐inhibitor group and the SPP1‐knockdown group compared to the negative control group (Figure 7I). These results from mice ESCA xenograft models showed that SPP1/JAK2/STAT3 pathway could strengthen radiation resistance in ESCA.

## 4 DISCUSSION

Esophageal cancer is the seventh most common cancer, but its' prognosis is poor.^1^, ^2^ Radiotherapy is one of the main treatment strategies to relieve symptoms and prolong patients' survival with ESCA,^5^, ^6^, ^7^, ^8^, ^54^, ^55^ while radio‐resistance often occurs during the treatment process and leads to treatment failure, which is a major obstacle in the treatment of ESCA.^9^ In this study, we identified a novel phenomenon that the SPP1/JAK2/STAT3 pathway is involved in the radiation resistance of ESCA, which may be an effective target for radiosensitization during the treatment process of ESCA.

In previous studies, SPP1 has been found to play significant roles in diverse biological processes such as biomineralization, bone remodeling, and immunological process,^12^, ^20^ and is correlated with multiple diseases such as autoimmune diseases, osteoarthritis, and inflammatory diseases.^56^, ^57^, ^58^, ^59^ More importantly, in the occurrence and development of tumors, SPP1 has been reported to be critical in tumorigenesis and tumor progression in many cancer types,^25^, ^29^ and the high expression of SPP1 is often correlated with poor prognosis.^24^, ^60^ Lin et al. have found the role of SPP1 in lung cancer evolution and heterogeneity and explored the potential of SPP1 as a therapeutic target.^52^ Katyana et al. have described the role (including proliferation, survival, migration, invasion, and angiogenesis) of SPP1 in colorectal cancer progression and outlined the interest in using SPP1 as a clinical biomarker.^53^

However, studies on the specific role of SPP1 in the development and progression of ESCA are few and not comprehensive and systematic enough. More importantly, there is no research on the relationship between SPP1 and radiosensitivity or radiation resistance so far. As for the current research on SPP1 in ESCA, Wang et al., Zhang et al. and Li et al. analyzed the prognostic value of SPP1 in ESCA through TCGA or other databases with no experiment verification.^36^, ^37^, ^38^ And to date, almost all studies on the prognostic and diagnostic value of SPP1 were based on the SPP1 levels in peripheral blood,^37^, ^61^, ^62^, ^63^, ^64^, ^65^, ^66^ while the SPP1 in the tumor microenvironment is of high levels and its effect on the tumor is theoretically more direct. In our study, we not only analyzed the prognostic and diagnostic value of SPP1 in multiple databases but also verified the results using clinical specimens, which made our results more authentic and reliable. We collected clinical tissue samples from patients with ESCA and verified the high expression of SPP1. Then we analyzed the relative expression level of SPP1 and patients' clinical information in our cohort, confirming that high SPP1 expression was negatively correlated with survival time in patients with ESCA. Indeed, our data are plausible because the prognostic results of SPP1 in ESCA in the TCGA database were consistent with our experimental data.

Since radiotherapy is a critical treatment for ESCA,^5^, ^6^, ^7^, ^8^, ^9^ we intended to investigate the relationship between SPP1 and radiosensitivity, which has not been studied so far. We found a novel phenomenon that the DNA damage repair ability of ESCA cells post‐radiation was weakened after SPP1 knockdown, this might be attributed to the decreased protein expression levels of pATM/TP53/p21/p27 post‐radiation, which led to cell cycle stagnation and helped DNA repair. When ESCA cells were irradiated, the expression of pATM/TP53/p21/p27 increased to stop the cell cycle so that the cells could repair the damaged DNA. However, when SPP1 was knocked down, the increase of pATM/TP53/p21/p27 post‐radiation was partially inhibited, which impeded the repair of DNA, and when SPP1 was overexpressed, the phenomenon was the opposite. What is more, we first uncovered that the pro‐apoptotic proteins such as Bax, cleaved‐caspase3, and FADD were increased after SPP1 knockdown, while the anti‐apoptotic proteins such as Livin and XIAP were decreased. To further verify the role of SPP1 in radio‐resistance in ESCA, we also analyzed the post‐radiation survival curves of ESCA cells using a single‐hit multi‐target model and found that the mean lethal radiation dose of ESCA cells increased after SPP1 knockdown. All these results suggested that downregulation of SPP1 could increase tumor cell death ratio and enhance radiation sensitivity in ESCA. What is more, by a comprehensive study with clinical samples, cell models, and animal models, we systematically report the onco‐promoting roles of SPP1 in ESCA. There are few studies on the function of SPP1 in ESCA, Jules Lin et al. found SPP1‐b‐type significantly enhanced ESCA cells' migration ability only by adding exogenous SPP1.^39^ In our study, we found that knocking down SPP1 reduced the proliferation, migration, and cell survival of ESCA cells, while overexpression of SPP1 increased these abilities, which is consistent with the tumor‐promoting effect of SPP1 in other tumors.^25^, ^52^ Each function was validated in two ESCA cell lines by multiple experiments.

To further explore the correlation between SPP1 and radio‐resistance, we tested the protein level of SPP1 post‐radiation and surprisingly found an increase. We also verified that the ability of DNA damage repair of ESCA cells post‐radiation was increased after SPP1 overexpression, and the mean lethal radiation dose of ESCA cells was also increased, indicating that overexpression of SPP1 could weaken the anti‐tumor effect of radiation and increase the radioresistance of ESCA cells.

Now that we have revealed the novel phenomenon that SPP1 promotes radiation resistance, we then intended to explore the specific downstream pathway through which SPP1 causes radio resistance. The JAK–STAT signaling pathway has been implicated to be involved in human cancer development, progression, metastasis, and resistance to treatments.^42^ Previous studies have uncovered that the JAK–STAT pathway is a downstream pathway of SPP1 in murine mammary epithelial tumor^48^ and breast cancer.^49^ However, there is no research on SPP1 and JAK–STAT pathway in ESCA so far. In addition, previous studies have confirmed that the JAK–STAT pathway is related to radio‐resistance in colorectal cancer and prostate cancer,^43^, ^44^, ^45^ while there are no studies focused on the correlation between JAK–STAT pathway and radiation in ESCA. To explore the relationship between SPP1 and JAK–STAT signaling pathway in ESCA, we conducted a Gene Set Enrichment Analysis between low and high SPP1 expression data using the TCGA database and found that the JAK–STAT signaling pathway had significant enrichment with SPP1 high‐expression phenotype. And we first found that the phosphorylation level of JAK2 and STAT3 increased, together with SPP1 increased post‐radiation in ESCA cells. Then we verified the phosphorylation level increase of JAK2 and STAT3 post‐radiation were mediated by SPP1. Our study has also proved that using STAT3 inhibitor could almost eliminate the radio‐resistance phenomenon induced by SPP1 overexpression, while STAT3 inhibitor could also strengthen the radiosensitivity like SPP1 knockdown in ESCA cells. In addition, we further confirmed our results in vivo with tumor xenograft models, which made our conclusion more credible.

All the above results suggest that SPP1 plays an important role in the radiation resistance of ESCA. Blocking SPP1 may help to reduce radiation resistance in ESCA patients, improving the therapeutic effect. Guangtong Deng et al. found that BET inhibitor indirectly inhibits SPP1 through NFKB in melanoma,^67^ but whether this inhibition also plays a role in other tumor cells is unknown. Unfortunately, there are no effective inhibitors of SPP1 with definite effects at present. The development of SPP1 inhibitors is very critical for alleviating radio‐resistance in ESCA patients, which will be a very important research focus in the future.

In summary, we reported the novel phenomenon that radiation could increase the phosphorylation level of JAK2 and STAT3 by increasing SPP1 expression and identified that the SPP1/JAK2/STAT3 axis is a critical player in ESCA progression and radiation resistance, which is a potential target for combined therapy to improve curative effect and increase patients' survival with ESCA.

## AUTHOR CONTRIBUTIONS

Meijie Wang was responsible for the main idea design, cell experiments, animal experiments, literature review, and article writing. Xiaozheng Sun and Huixian Xin were responsible for part of the literature review. Yufeng cheng and Zhihua Wen provide funding support and part of the article's idea.

## CONFLICT OF INTEREST

The authors have no conflict of interest.

## ETHICAL STATEMENT

All the protocol for research was approved by the Medical Ethical Committee of Shandong University Qilu Hospital and it conforms to the provisions of the Declaration of Helsinki. Animal research was approved by the Animal Ethical Committee of Qilu Hospital of Shandong University.

## Supporting information


Figure S1

Figure S2

## Data Availability

The data that support the findings of this study are available from the corresponding author upon reasonable request.
