# Supplementary figures and images for: SPP1 promotes radiation resistance through JAK2/STAT3 pathway in esophageal carcinoma
Source: Cancer Med. 2022 May 20;11(23):4526–43. doi: 10.1002/cam4.4840 (PMC9741975; doi:10.1002/cam4.4840)

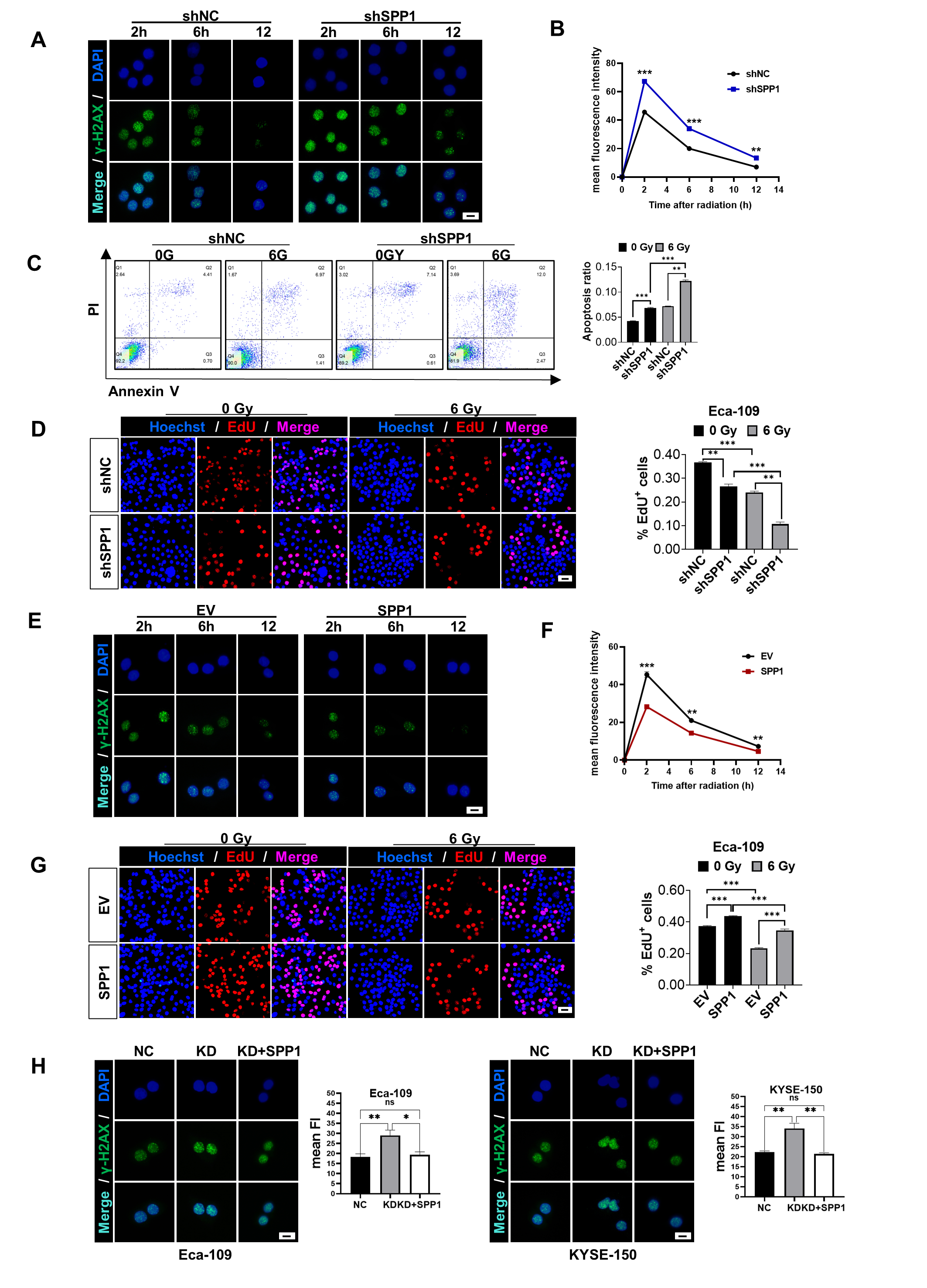

Supplement: Supplementary file 1 — Figure S1 [file CAM4-11-4526-s002.TIF]

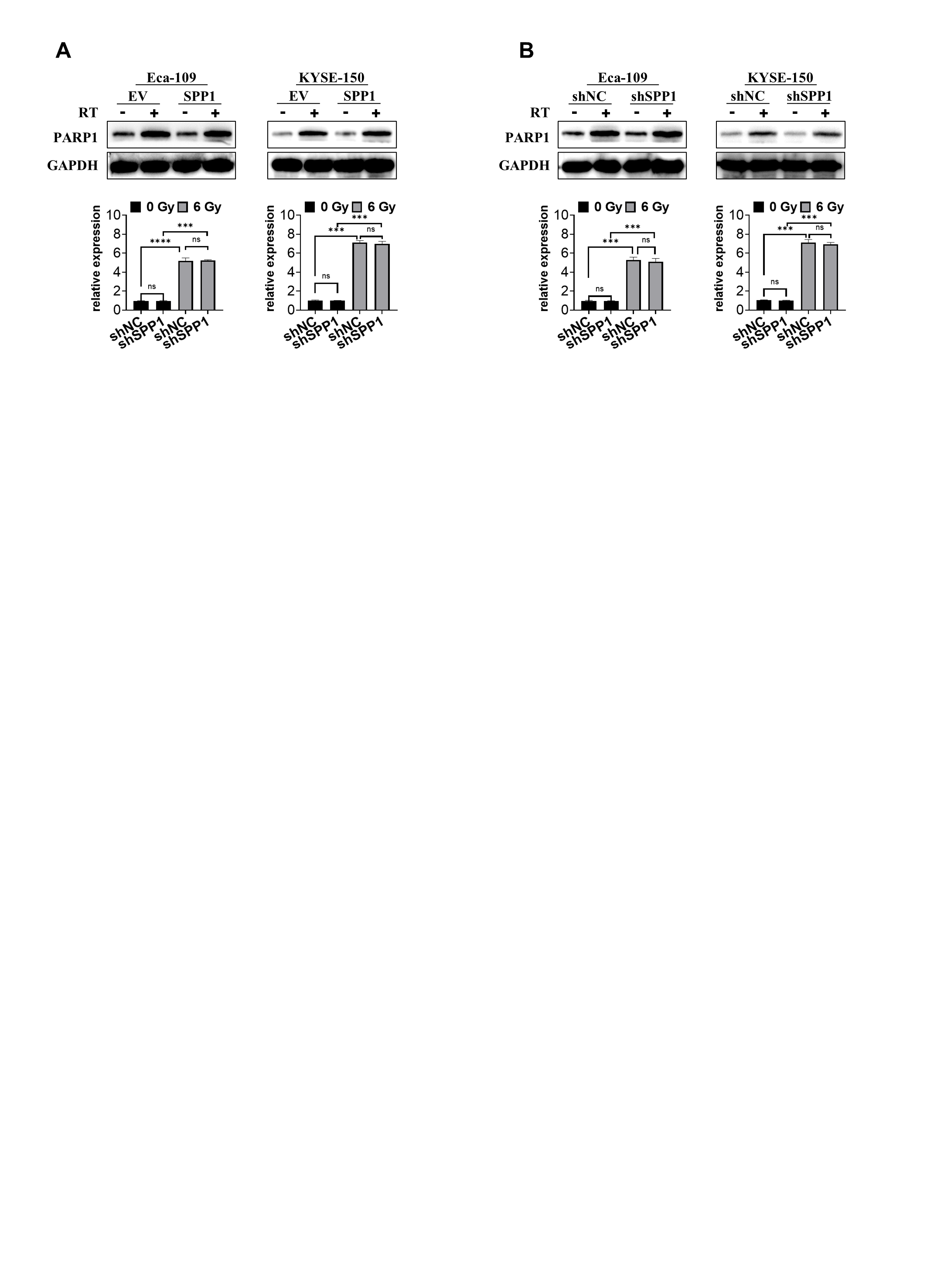

Supplement: Supplementary file 2 — Figure S2 [file CAM4-11-4526-s001.TIF]
